# Integrated vector control of Chagas disease in Guatemala: a case of social innovation in health

**DOI:** 10.1186/s40249-020-00639-w

**Published:** 2020-04-14

**Authors:** Diana Castro-Arroyave, Maria Carlota Monroy, Maria Isabel Irurita

**Affiliations:** 1 Centro Internacional de Entrenamiento e Investigaciones Médicas, CIDEIM, Cali, Colombia; 2 Universidad Icesi, Cali, Colombia; 3 Laboratorio de Entomología y Parasitología Aplicada (LENAP), Facultad de Farmacia. Universidad de San Carlos de Guatemala (USAC), Guatemala City, Guatemala

**Keywords:** Chagas disease, Community empowerment, Interdisciplinarity, Intersectorality, Social innovation in health

## Abstract

**Background:**

Improved access to health care and quality of services require integrated efforts and innovations, including community empowerment and participation in transformation processes. Chagas disease is a neglected tropical disease that is generally controlled by insecticide spraying. To achieve community empowerment in a health program, actions for social innovations may include: community-based research, interdisciplinary and intersectoral participation, community perception of direct benefits and participation in health or environmental improvements. The aim of this study was to describe and analyze the processes by which an interdisciplinary team, in collaboration with communities of Comapa, Guatemala, developed an effective solution to address the risk for Chagas disease.

**Methods:**

A qualitative study involving interviews semi-structured and direct observation was conducted using a case study approach to describe and understand the community-based research and intervention process developed by researchers from the Laboratory of Applied Entomology and Parasitology of the Universidad de San Carlos of Guatemala (Laboratorio de Entomologia y Parasitologia Aplicada). Nine interviews were conducted with the investigators, innovators, members of the community in which the intervention had been implemented. NVivo software (version 12) was used for the emergent coding and analysis of the interviews.

**Results:**

Processes of social transformation were evident within households, and the communities that transcended the mere improvement of walls and floors. New social dynamics that favored the household economy and conditions of hygiene and home care that positively impacted the health of the community. We describe how the integration of criteria of social innovation into a home improvement strategy for Chagas disease control, can generate processes of transformation in health by considering sociocultural conditions, encouraging dialogue between public health approaches and traditional practices. We identify and discuss processes for Social Innovations in Health and identify their potential in improving community health in Latin America.

**Conclusions:**

When social innovation criteria are included in a health control initiative, the community-based research and the interdisciplinary and intersectoral participation facilitate the implementation of the control strategy, the perceived benefits by the community and its empowerment to sustain and share the strategy. The case study provided understanding of the intersectoral and interdisciplinary dynamics in particular contexts, and documented the relevance of innovation criteria in health processes.

## Background

Significant gaps in access to and quality of health services, associated with climate change, violence, and growing inequalities between and within regions and countries challenge the global community. In low- and middle-income countries, additional burdens exist, including those associated with diseases of poverty like HIV/AIDS, tuberculosis, malaria, and leishmaniasis and Chagas disease, among others. More than one billion people worldwide are estimated to be affected by such diseases; these are usually, according to Ensor [1], poorly researched and inadequately understood. The World Health Organization (WHO) has classified approximately 20 of these conditions as “neglected tropical diseases,” which neither attract the attention of global media nor receive the funding required to address them. Chagas disease, also known as American trypanosomiasis, is one of these neglected diseases that contributes to continuing poor health and poverty in the American region.

The disease was first described by Brazilian physician Carlos Chagas in 1909. One hundred and 10 years later, it remains one of the most economically devastating parasitic diseases in Latin America. This chronic disease affects about six million people and some 65 million people are at risk of contracting the disease [2]. The annual incidence is estimated at 28 000 cases, and 12 000 deaths per year.

Chagas disease is a zoonosis that is strongly associated with poverty in rural Latin America. Houses made of adobe or plant material, common in rural Latin America, provide a perfect habitat for triatomine bugs, the vectors of Chagas disease. Triatomines are obligate blood feeders at each stage of their lifecycle, and transmit the flagellated protozoal Chagas parasite, *Trypanosoma cruzi*. While feeding, triatomines ingest a large quantity of blood and simultaneously defecate parasite-containing feces that are the source of infection via contamination of the wound caused by the insect bite or of ocular or oral mucosa.

The geographic distribution of the diverse vector species ranges from Mexico to the Southern cone of South America [3]. In Guatemala, communities within 21 of the 22 national departments (states) present triatomines inside of homes in rural and periurban communities [4]. The main risk factors are crevices in the adobe walls, permanent presence of animals inside the houses, and dirt floors [5, 6]. Other factors include lack of plumbing facilities and use of thatched roofing and age of the house. Additionally, colonization of houses by vectors increases with deforestation and changes in land cover [7]. The borders of Guatemala with El Salvador and Honduras, where the ethnic communities of Chorti, Xinca, Lenca and Ladino dominate, are highly endemic [8].

In Mesoamerica, the elimination of *Rhodnius prolixus* through regular application of insecticides [9] was followed by replacement by another species, *Triatoma dimidiata* as the main vector of Chagas disease. This is a complex of species, and the different genetic lineages may explain different epidemiologic relations and responses to control efforts within the region. For maximum public health impact, the design of a control strategy that fits or is applicable to all genetic lineages is desirable.

*Triatoma dimidiata* is a native species (originated in the region) [10] and endemic vector that is in the process of domiciliation (adapting to and colonization of human environments), while also living and propagating in sylvatic (forest) conditions [11]. From 2000 to 2008, the Government of Guatemala pursued a massive insecticide campaign, but the species still invades and colonizes houses, presenting infestation indexes between 3 and 35% of houses [12, 13]. Where *T. dimidiata* is not strictly domesticated, insecticide spraying has been ineffective [12] or needs to be repeated frequently due to re-invasion of insects from the sylvatic cycle in nearby forests [13]. However, repeat applications are beyond the resources of many affected countries, and insecticides can cause environmental damage. *T. dimidiata* readily re-infests insecticide-treated houses. Consequently a new approach was needed for a cost-effective, environmentally safe, long-term reduction of house infestation [13]. The social innovation that we report here, was designed to involve community participation, address risk factors, with the support of interdisciplinary teams and intersectoral participation in an integrated control strategy. The goal was to ensure healthier living conditions in which Chagas disease control would be an important benefit.

The complexity of Chagas disease demands actions that effectively integrate interdisciplinary and intersectoral approaches that build applied knowledge taking into consideration the sociopolitical, economic and cultural contexts of diverse communities and social groups. Based upon this principle, and targeting health situations that afflict low- and middle-income populations worldwide, a Social Innovation in Health Initiative (SIHI) was conceived and launched in 2015. This initiative was constituted as “a global network of individuals, organizations and institutions advocating for social innovation in health and advancing research in social innovation” [14]. The initiative currently has five hubs across continents. The Latin American and the Caribbean region Hub (SIHI-LAC-Hub) is constituted by a consortium including Centro Internacional de Entrenamiento e Investigaciones Médicas (CIDEIM), Pan-American Health Organization (PAHO) and the Universidad Icesi, headquartered in Colombia. This and other SIHI Hubs identify and promote social innovation solutions in health, and strengthen communities of stakeholders locally and regionally.

Social innovation for some decades, occupied an important place in the search for solutions to problems related to climate change, the global epidemic of chronic diseases, and increasing morbidity and mortality due to diseases associated with poverty and increasing social inequality [15]. However, the theoretical and methodological developments in this area have focused more on entrepreneurship and technological innovation than on the applied vision of social innovation as a means of supporting community empowerment and improving access to and quality of health care. As stated by SIHI Global, the vision of social innovation acts as a novel lens whereby provision of health services can be approached from a systems perspective, thereby enhancing the development of solutions by individuals and institutions from different sectors and disciplines, through involvement and commitment of communities [16].

Within the framework of SIHI-LAC-Hub, CIDEIM, Universidad Icesi and PAHO developed a call to identify social innovation initiatives in health in Central America in 2017. The Comapa project was among the projects identified through this crowd-sourcing initiative. Using a case study methodology, the aim of this study was to explore and describe the processes by which an interdisciplinary team, in collaboration with rural communities of Comapa, Guatemala, developed an effective solution to address the risk for Chagas disease. As we describe, this solution led to the emergence of interdisciplinarity, intersectorality and empowerment, as a result of the intervention process and as an important methodological component to support social transformation.

### General description of the case

A research group from Laboratory of Applied Entomology and Parasitology (LENAP) of the Universidad de San Carlos (USAC) developed an effective and innovative social approach for the control and prevention of Chagas disease in the municipality of Comapa, Guatemala. The approach consisted in designing a strategy to address predetermined risk factors for the colonization of dwellings by the vectors. The interventions included filling the cracks and crevices in the floors and walls using a combination of locally available materials, raising awareness and training of leaders and members of the community to adopt the home improvements and contribute to cultural changes such as maintaining animals outside homes to eliminate the risk of colonization of homes by triatomine vectors [6, 17].

The eco-health approach to Chagas disease created social value by effectively reducing vector infestation rates, and by reducing the levels of infections, illnesses and deaths in the regions where it has operated [18]. It also contributed to positive cultural and behavioral change in the communities that it impacted. In addition, the improvement changed the appearance and quality of the household environment, leading inhabitants to make further improvements and undertake initiatives such as planting decorative plants and trees for fruit cultivation [6, 17].

## Methods

Qualitative research was conducted using a case study approach to investigate and understand the community-based research and intervention process developed by researchers from the Laboratory of Applied Entomology and Parasitology of the Universidad de San Carlos de Guatemala. As Martinez [19] points out, citing Eisenhardt (1989), the case study as a method provides a strategy to understand the dynamics present in particular contexts, and allows the collection of information from different perspectives.

### Data sources

Direct observation and semi-structured interviews were used to gather qualitative data that required direct contact with communities, contexts and specific realities. Data were collected through the field notes of two social researchers. Interviews were audio-recorded during visits to USAC and the participating communities of Comapa. Other sources included documents about Comapa and Chagas disease, and publications produced by the researchers responsible for the Guatemala initiative.

Nine interviews seeking insight into the problem for which the control strategy was designed, the role of the community and partner institutions in the design and implementation, and innovative features of the initiative. The results of the experience and the projection of the strategy within other communities and the health system. The interviews of approximately 1 h each were conducted with men and women, 18 years of age and older, who were residents of the four communities of Comapa in which the intervention was developed. People from academic and government sectors were also interviewed. All participants were involved in the housing improvement activities to reduce triatomines in homes and communities, which was an important criterion for choosing the participants in the case study. Snowball and theoretical samples were used to recruit participants.

### Data analysis

The interviews and field notes were transcribed verbatim and were manually analyzed to achieve a level of organization of the information sources accessible to the research team. In a second phase, NVivo 12 (QSR international https://www.qsrinternational.com/nvivo/home) was used for the emergent coding of the nine interviews. This information was organized according to the three categories of analysis reflected in this article: *empowerment, interdisciplinarity* and *intersectorality*. The approach to analysis followed that offered by Strauss and Corbin [20] in the development of grounded theory.

### Ethics

The case study protocol previously developed by SIHI was translated and adapted to the Latin American context, and subsequently reviewed and approved by the CIDEIM institutional Ethics Committee for Research in Human Subjects. The study was explained to the interviewees and study participants signed an informed consent to ensure the voluntary nature of their participation, their right to withdraw at any time, and the protection of their identity. Written authorization to audio-record the interviews was provided separately.

## Results

All individuals interviewed reported that the strategy of home improvement achieved the articulation of public and private institutions with the community. For the participants in this case study, it was evident that the new conditions of the households motivated women and their families to adopt new and health promoting practices that also favorably impacted household and community economies. Planting and cultivation of fruit trees, commercialization of the fruit produced and the change in management practices of domestic animals contributed to the quality of life of the members of the participating communities.

### Community-based research

The communities of Comapa, Guatemala, like many others in Latin America, face health challenges associated with gaps and limitations in access to and the quality of health services. The knowledge acquired by researchers from University of San Carlos (USAC) in Guatemala about how to improve houses with local material, to avoid the colonization by triatomine bugs that transmit Chagas disease, gave rise to the need to transcend the traditional vision of research and to move towards a perspective that involves the community, promoting their empowerment and participation. This move enabled researchers to understand the reality of families affected by or at risk for Chagas disease, and to design interventions in a collaborative, integral and contextualized manner.

Recognition of this need and the potential of the intervention to address the problem, motivated researchers, innovators and local community members to work closely in a coordinated manner. The steps followed in this process were first to analyze and understand the context which resulted in risk factors for acquiring Chagas disease, and then to identify and prioritize those risk factors that could be addressed within and by the community, which might both reduce the risk of Chagas disease and other diseases with similar risk factors.

The LENAP researchers of the Universidad de San Carlos consulted community members about everyday activities and practices that constituted risk factors of infection, and identified possible actions to reduce them. They proposed and negotiated ways to carry out housing improvement plans with the participation of men and women from the communities. During this process, the definition of materials, uses and customs that could contribute to the objectives of the intervention process was key.

The housing improvement strategy and other components of the intervention in the field were then implemented and evaluated. This test provided visibility to the changes that the intervention generated in the homes and in the daily lives of communities, and provided the bases to replicate, implement and scale up the innovation in neighboring countries including El Salvador, Honduras and Nicaragua.

### Interdisciplinarity as integral to the initiative

Inter-disciplinarity was both an input, a methodological approach and a tangible result of this effort to reduce the presence and incidence of Chagas disease. LENAP was established in 1992 primarily with biologists and microbiologists from Universidad de San Carlos. The first national attempts to control Chagas disease involved insecticide spraying, at a time when, inside the laboratory, the research team was trying to understand the epidemiology of transmission in the affected communities. Adobe housing typical of poor rural communities was recognized as creating the conditions for the colonization of the houses by the triatomine bugs, and environmental factors such as the introduction of Eucalyptus trees in replacement of native woods precipitated the migration of the vector insect from forests into the houses.

Prior to the development of the integrated control strategy described in this case study, the LENAP team identified risk factors associated with the presence of the insects, using ecological and statistical expertise [5]. Three risk factors in particular, were able to be modified and hence were targeted for eco-health interventions: the crevices in the adobe walls, the cracks and holes in mud floors, and the custom of rearing and permanently housing animals inside the households [6].

The use of epidemiological evidence to define an intervention strategy with the community marked a turning point in the approach to the problem. As biologists, the researchers knew little about how to improve floors or walls, and this led to their collaboration with architects and engineers. As biologists, the researchers knew little about how to improve floors or walls, and this led to their collaboration with architects and engineers. In 2004, the first home improvements were undertaken to address the quality of floors and walls, and these early interventions were shown to reduce the prevalence of triatomine bugs. Subsequently anthropologists, sociologists and others became involved in the educational component of the home improvement initiative. Veterinarians also assisted with infected pets (mainly dogs) and in guiding the relocation and management of corral animals to outdoor locations.

Students who were undertaking internships with LENAP also contributed, as one student described: “I came here because I wanted to learn about molecular biology and genetics and I did, but I learned also about human and sociological perspectives, approaches that I had not considered before” (biology student, Comapa, 2017).

The willingness of the team to encourage open dialogue among include different disciplines and social perspectives was also useful to establish trust and empathic relationship with the communities. Community members participated as active subjects of the social innovation in health solution and contributed their practical social and cultural knowledge as home improvements began to be undertaken. When introducing the intervention, the team needed to use a simple and contextualized language to communicate with community members.

Early in the development of this initiative, interdisciplinary dialogue had been highly encouraged and promoted by the international funders of the housing intervention but it soon became the manner through which the researchers conceived their work dynamic. Inter-disciplinarity, as well as being a strategy, became a way of doing things, and was, in this case, an important result of the process of responding to a health situation from a comprehensive perspective. Seven architecture and engineering theses were written, and the theses suggested that researchers needed to explain to community members how to choose suitable material, help them build, and design more field tests rather than conduct laboratory research.

### Intersectorality as a condition to improve the health of the communities

The eco-health approach (based on environmental, social and biological risk factor management) described here is intersectoral as well as interdisciplinary. From the early days of LENAP, the team found financial backing to deploy their program through international donors. International Development Research Centre (IDRC) of Canada, funded the development of the innovation and supported the scale up to El Salvador and Honduras (2011); the Japanese International Cooperation Agency (JICA) funded the transfer of the program to Nicaragua (2014).

Over time, other key alliances were also established. The Ministry of Health of Guatemala, international non-government organizations (NGOs), and local and regional agencies joined the initiative, thus boosting positive impact on triatomine bug control and reduction of Chagas disease. The articulation of organizations from public and private sectors was recognized and valued by researchers, innovators and communities as positive and collaborative strategy. Organizations, including the Ministry of Health and its officers, recognized that the benefits of implementing this initiative outweighed the supposed benefits of conventional chemical sprayings.

When a new geographical area was prioritized and scheduled for a home improvement intervention, the team at LENAP established contact with a team from the national program for the prevention of Chagas disease to ensure that actions and field visits coincided. Coordination was not always easy, as it was difficult to obtain full participation from the municipalities, but some were supportive.

International NGOs with presence in several countries joined the initiative to work with children and with mothers on health and education issues, and the team at LENAP partnered with them to include in the workshop awareness of personal and family hygiene and the need to keep homes free from bugs. Health education took a leading role and shared the stage with basic research to promote processes of social transformation in health.

Local politicians also joined the initiative. In this regard, a participant in the case study said: “We try to collaborate with help the university because none of us can tackle this problem alone” (local politician, Comapa, 2017). In Comapa, the team from USAC supported a functional network that included the local health center, with staff trained by the team to educate people treated for Chagas disease and to test pregnant women for infection with *Trypanosoma cruzi*.

The different institutions and sectors worked together to define individual roles and responsibilities, contributing to the effectiveness of the partnerships and the fulfillment of the objectives of the initiative. Overall, the team at LENAP orchestrated the home improvement strategy in rural areas and conducted the laboratory tests, the Ministry of Health continued spraying and providing treatment, while staff at the health center obtained blood samples that are transported to a laboratory, and continuously monitored patients for symptoms of illness. The Mayor’s office provides the transportation of local materials for house improvements in the villages.

“For us as representatives of the Ministry of Health of Guatemala and as health workers, universities have been very helpful. They have come to strengthen the surveillance system of our municipalities. Together we can do things, but alone it is more difficult” (official of the Ministry of Health, Guatemala, 2017). This testimony illustrates the importance of interaction and collaborative work among institutions and sectors, including academic and government sectors, as represented by researchers and decision makers.

Intersectionality and interdisciplinarity are in this case a necessary condition for future interventions to ensure the replicability, scalability and sustainability of the initiative.

### Community empowerment in social innovations in health

University-based researchers do not always recognize that individuals and communities that participate in a study are social actors with the capacity to contribute to the processes of social transformation and the development of actions to achieve the objectives. The tendency to see community members as objects of study and not as interacting agents with important contributions in the construction of knowledge remains a challenge in research and in the training of researchers.

The main objective of this innovation was the control of the vector responsible for the transmission of Chagas disease. In this process, the role of communities and the contextualized reading of their social and cultural practices was fundamental.

“I knew I had to make the house strong, but I could not do it alone” (researcher, Comapa, 2017). That is, the researchers clearly knew what changes were necessary to avoid the disease, but it was necessary to translate the idea to communities. Researchers need help and commitment from community leaders and different social groups. This testimony offers evidence of the moment in which the researcher, despite being the one who generated the idea of improving housing, with the knowledge and experience in the laboratory with the vector and Chagas disease, recognizes the constraints of working alone and the need for wide intersectoral involvement. Local institutions and residents know their homes, their environment, their needs and resources to work on housing improvement, while preserving and respecting cultural aspects such as the absence of windows in buildings and the types of material available in the community to fill crevices and cracks in walls and floors. *“*Getting the community involved is important, since they are responsible for their lives and have knowledge and responsibilities to examine the walls and capture the vector, but mainly, they are the ones who should prevent the bug from continuing to colonize their homes” (member of fieldwork team, Jutiapa, 2017). In this testimony, responsibility and participation are presented as key to achieving the objectives, to educate communities in health, and to work with them. The respect of local contexts and sociocultural realities gave rise to ownership and community engagement, and enabled social empowerment and the social appropriation of knowledge and solutions.

Housing improvement brought about a series of changes in the practices and lifestyles of the communities: both community and gender empowerment were unexpected results, not foreseen by the researchers. As a result of the visible improvements to houses, families and groups of women began to organize areas outside their houses, as one participant explained:

As we put a lot of emphasis on removing animals from the houses, people started to worry more about the outside of their homes. We have noticed, for example, that many of them planted trees outside the houses. In addition, many women started to grow fruit trees [...] now they are selling mango, papaya, nances (local fruit), lemons and other fruits that they had not produced before. We can say that the empowerment of women, economic independence and better use of land were also the result of improving housing (researcher and innovator observations, Guatemala, 2017).

By reducing the presence of the vector and the risk of Chagas disease in the intervention areas, the eco-health approach created social value in its most evident form: saving lives from preventable deaths. At the same time, the approach strengthened the capacity of these communities to solve a range of problems, not just health-related ones. An integral and complex intervention made through home improvement, focusing on floors and walls, with its capacity to eradicate the presence of the bugs, inspired other interventions that enhanced the quality of life and the well-being of the people.

The promotion of health was a fundamental approach in the improvement of the health of participants.

## Discussion

In the above case, the reduction of infestations of triatomine bug in improved houses was demonstrated in Guatemala [17, 21]; Polymerase chain reaction (PCR) techniques allowed the researchers to evaluate changes in the bug’s food source after housing improvement, thereby confirming a reduced risk of human-vector contact [17]. Chagas disease could therefore be prevented in communities where most of the houses are improved, eliminating the need for insecticide because the houses were resistant to invasion of the bugs [22]. After the improvements the triatomine bugs were unable to colonize the houses, resulting in feeding on extradomiciliary animals, especially birds [17], thereby diminishing feeding on humans and decreasing *Trypanosoma cruzi* infection, since birds are refractory to infected by this parasite [17].

Infestation rates decreased dramatically inside homes and as long as the walls were kept smooth and without crevices, the triatomine bug was unable to establish itself and reproduce within the households. Spatial analysis of the before and after distribution of vectors [21] substantiated this change.

Eighty percent of triatomine bugs were collected in cracks and crevices in walls [23], and wall plastering took place before improvements to the dirt floor and changes in animal management [24]. Families received training and materials (volcanic ash and lime from nearby areas) to undertake house improvement; weekly monitoring visits enabled any corrections to the process or increased supply of materials. The municipality helped supply the volcanic ash (used also in road construction), and personnel in the Ministry of Health learned the procedure and helped in monitoring. The cost of improvements was ten times less than traditional use of cement, making sustainability easier. Although traditionally, women applied wall plaster using only one material (mud), teaching them to use a mixture of materials that made walls resistant to bugs and was sustainable, was found to be easy. Consequently empowerment was not difficult. This form of empowerment in the communities transcended the objective of improving housing, and was reflected in other activities such as entrepreneurship and the economic autonomy of women through cultivation, management of the chickens, and marketing fruits.

The initiative began in four villages [8] and was later scaled up to more than 17 villages in three different countries with diverse ecosystems and ethnic populations [24, 25]. Scaling up was shown to be possible in diverse epidemiological conditions [25]. Including activities such as native tree nurseries and the technical management of stingless bees was easier in communities once they saw the value of those activities, illustrating the importance of taking into account contextual and socio-cultural characteristics when implementing an initiative as a social innovation in health. The construction of wire chicken coops and the technical management of birds by women in some areas increased the protein intake of householders and provided women with an income [9], and gave rise to further interdisciplinary and inter-institutional dialogue with a high degree of community involvement.

In the search for an effective solution to a specific health problem, three processes emerged, giving shape to this experience and contributing towards interdisciplinarity, intersectorality and community empowerment. These three processes generated a multidisciplinary research team of dynamic partners in governmental, NGO agencies, academia and the community. These processes were not just methodological choices and outcomes of an eco-health approach, but will also be crucial to future social innovations in health.

Many community members began improving their surroundings as an immediate result of the household interventions to prevent infestation by triatomine bugs, the improvements included looking after their plots and motivation to productively farm. This increased women’s income and engaged many who had not previously participated in agricultural activities. With higher income came better nutrition, and more plantations around the houses, including trees providing shade, and cooler households. These factors contributed to improved health and wellbeing [26].

This innovative program always engaged local promoters, young men who were trained as facilitators and field technicians for the home improvement program. They became advocates of this approach in their own communities and in other places they visited, under the coordination and with the accompaniment of LENAP. The program instilled a sense of capacity and promoted community empowerment. This in turn attracted other resources to the communities.

Three limitations of this case study should be recognized. First, the period of time for researchers to learn about the initiative and conduct interviews with the communities and other partners was short. Second, the household improvement experience for the control of Chagas disease has been transferred to other countries, but in this case study only the Guatemala initiative was considered - therefore these results may not be generalizable to other contexts. Third, the researchers/authors recognize that evaluation of the cost-benefit relationship of the intervention could contribute to the replicability and sustainability of social innovation in health initiatives.

## Conclusions

When the search for a solution to a health problem involves more than health specialists, it is likely to address certain underlying structural causes that create the social, cultural and economic conditions for a problem to prevail. Interventions designed from an interdisciplinary and intersectoral perspective are likely to initiate lasting processes of social transformation with the capacity to involve communities as the main architects of their own progress. Community empowerment is a process, a result and an effect of the home improvement program that is demonstrating that Chagas disease can be a disease of the past.

Since the eco-health strategy was strengthened when stakeholders interact, the efforts of researchers and communities benefit when national and international organizations work together in communicating the effectiveness of, and in implementing this new approach to the control of Chagas disease. Other stakeholders and actors in replicating the approach in different contexts can be convened to achieve tailored intersectorial and interdisciplinar partnerships to improve health and quality of life.

## Data Availability

Not applicable.

## Abbreviations

AIDS: Acquired immune deficiency syndrome
CIDEIM: Centro Internacional de Entrenamiento e Investigaciones Médicas
HIV: Human immunodeficiency virus
IDRC: International Development Research Centre
JICA: Japanese International Cooperation Agency
LENAP: Laboratory of Applied Entomology and Parasitology
PAHO: Pan-American Health Organization
PCR: Polymerase chain reaction
SIHI LAC Hub: Latin American & the Caribbean Hub
SIHI: Social Innovation in Health Initiative.
TDR: Special Programme for Research and Training in Tropical Diseases, cosponsored by the United Nations Children’s Fund, the United Nations Development Programme, the World Bank and the World Health Organization
UNDP: United Nations Development Programme
UNICEF: United Nations Children’s Fund
USAC: Universidad de San Carlos
WHO: World Health Organization
